# The DEAD-Box RNA Helicases of *Bacillus subtilis* as a Model to Evaluate Genetic Compensation Among Duplicate Genes

**DOI:** 10.3389/fmicb.2018.02261

**Published:** 2018-09-25

**Authors:** José Antonio González-Gutiérrez, Diana Fabiola Díaz-Jiménez, Itzel Vargas-Pérez, Gabriel Guillén-Solís, Jörg Stülke, Gabriela Olmedo-Álvarez

**Affiliations:** ^1^Departamento de Ingeniería Genética, Unidad Irapuato, Centro de Investigación y de Estudios Avanzados del Instituto Politécnico Nacional, Guanajuato, Mexico; ^2^Departamento de Biología Molecular de Plantas, Instituto de Biotecnología, Universidad Nacional Autónoma de México, Cuernavaca, Mexico; ^3^Department of General Microbiology, Institute of Microbiology and Genetics, Georg-August-Universität Göttingen, Göttingen, Germany

**Keywords:** *Bacillus subtilis*, duplicate-gene, gene–gene interactions, epistasis, DEAD-box RNA helicases

## Abstract

The presence of duplicated genes in organisms is well documented. There is increasing interest in understanding how these genes subfunctionalize and whether functional overlap can explain the fact that some of these genes are dispensable. *Bacillus subtilis* possesses four DEAD-box RNA helicases (DBRH) genes, *cshA, cshB, deaD/yxiN*, and *yfmL* that make a good case to study to what extent they can complement each other despite their subfunctionalization. They possess the highly conserved N-terminal catalytic domain core common to RNA helicases, but different carboxy-terminal ends. All four genes have been shown to have independent functions although all participate in rRNA assembly. None of the *B. subtilis* DBRH is essential for growth at 37°C, and all single deletion mutants exhibit defective growth at 18°C except for Δ*deaD/yxiN*. Evaluation of double mutants did not reveal negative epistasis, suggesting that they do not have overlapping functions. The absence of any one gene distorts the expression pattern of the others, but not in a specific pattern suggestive of compensation. Overexpression of these paralogous genes in the different mutant backgrounds did not result in cross-complementation, further confirming their lack of buffering capability. Since no complementation could be observed among full sized proteins, we evaluated to what extent the superfamily 2 (SF2) helicase core of the smallest DBRH, YfmL, could be functional when hooked to each of the C-terminal end of CshA, CshB, and DeaD/YxiN. None of the different chimeras complemented the different mutants, and instead, all chimeras inhibited the growth of the Δ*yfmL* mutant, and other combinations were also deleterious. Our findings suggest that the long time divergence between DEAD-box RNA helicase genes has resulted in specialized activities in RNA metabolism and shows that these duplicated genes cannot buffer one another.

## Introduction

In bacteria, up to 44% of genes could come from duplication (Zhang, 2003; Hannay et al., 2008; Serres et al., 2009). Most of the duplicated genes appear to result from gene duplication events, probably in response to different selection pressures, such as starvation conditions and physical stress. However, it is unclear how duplicate genes transit from an initial state of redundancy to a situation in which both copies are maintained, and an epistatic relationship is established. Redundancy is frequently associated with paralogs that share an identical biochemical function (Prince and Pickett, 2002). It explains a buffering relationship where genes can compensate for each other’s loss by their ability to share and takeover the same function. In this manner, genetic buffering results in the masking of the phenotypic consequences of mutations (Hartman et al., 2001; Thomaides et al., 2007). As DeLuna et al. (2008) suggest “functional overlap between paralogs explains the individual dispensability of most genes that are duplicated in *Saccharomyces cerevisiae.*"

**FIGURE 1:**
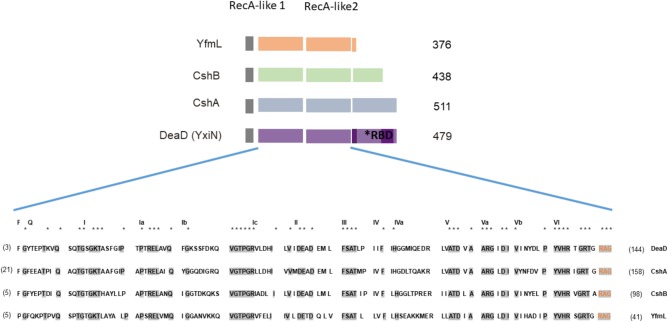
DEAD-box RNA helicases (DBRHs) of *Bacillus subtilis*. Diagram illustrating the conserved SF2 RNA helicase core (RecA-like 1 and RecA-like 2). The size of each helicase is shown. The alignment shows high conservation of sequence in the SF2 core for the four *B. subtilis* DEAD-box RNA helicase proteins. **Supplementary Table S6** shows the percent identity between the different DBRH core domains. The consensus sequence is highlighted in gray. The numbers in parenthesis represent the amino acid residues of the carboxy-terminal end. For the construction of the chimeras, the N terminal region used from YfmL extended from residue 1 to 335, including residues labeled in orange (RAG) conserved in all DBRH.

One of the most ancient and largest families of enzymes belongs to the superfamily 2 (SF2) of helicases, DBRH. The initial classification of the RNA helicases was done by Gorbalenya and Koonin (1993), on the basis of different conserved motifs in the amino acid sequence. Six superfamilies are distinguished, SF3 to SF6 form oligomeric rings while SF1 and SF2 do not. SF1 and SF2 contain two highly similar helicase domains in tandem, within a 300 amino acids region, but can be distinguished by variations within their conserved motifs (Anantharaman et al., 2002; Cordin et al., 2006). SF2 helicases, involved in virtually all aspects of RNA metabolism, share a catalytic core with high structural similarity, but different enzymes perform a wide spectrum of distinct functions on diverse substrate (Gorbalenya and Koonin, 1993; Bourgeois et al., 2016). Most SF2 family members are believed to be monomeric, but some have shown to be homodimeric and to possess a dimerization motif (Klostermeier and Rudolph, 2009). The SF2 catalytic core consists of two RecA-like domains that bind ATP and single-stranded RNA (Singleton et al., 2007) (**Figure 1**), while a flexible linker allows for different juxtapositions of the RecA-like domains. The name “DEAD box” comes from one of the conserved motifs within the helicase C-terminal domain, RecA-like 2. The DEAD box motif has variations among different members, so it is also referred to as DEXD box. The common structural features of the DEAD-box helicase core suggest that members of this family couple conformational changes to duplex destabilization and strand separation by a common mechanism. DEAD-box mediated RNA unwinding occurs when binding of RNA and ATP triggers a conformational change of the helicase core, that brings it to a close conformation, leading to a deformation of the RNA backbone and destabilization of the RNA duplex. A flexible linker allows for different juxtapositions of the RecA-like domains (Rudolph and Klostermeier, 2015). However, duplex unwinding is not always the primary physiological function, and there are significant mechanistic differences even for helicases that unwind duplexes (Jankowsky and Fairman, 2007). Flanking domains function in protein binding for recruiting the helicase to its cellular substrate, and also mediate either specific interaction with RNA or, more often, unspecific interactions to tether the helicase core to its target in a flexible manner, since some act on hundreds of different substrates. Therefore, DBRH are believed to be modular proteins combining the catalytic SF2 core with a C-terminal end that can bind proteins and RNA (Karginov and Uhlenbeck, 2004). Kossen et al. (2002) showed that the C-terminal domain of YxiN/DeaD could by itself bind RNA and the catalytic core could conserve its ATPase activity without the C-terminal end. The domain of the *B. subtilis* DEAD-box helicase YxiN/DeaD that is responsible for specific binding of 23S rRNA has an RNA recognition motif (RRM) fold (Wang et al., 2006). Its structure has been determined (Wang et al., 2006) and reveals an RRM comprising a central b-sheet, flanked by two a-helices on one side. Substrate specificity has been shown for this RNA helicase, as well as for the *Escherichia coli* RNA helicase, DbpA and *Thermus thermophilus* Hera. These proteins bind and unwind a helix adjacent to a stem-loop structure in 23S rRNA via RRM (Diges and Uhlenbeck, 2001; Linden et al., 2008). This substrate specificity is not present in all DBRH, and most exhibit non-specific binding of RNA via basic C-terminal domains and act as general chaperones. In this case, positively charged unstructured regions provide binding sites for structured RNA, for example CYT-19 (Busa et al., 2017). In most DEAD-box proteins, the helicase core is flanked by N- and C-terminal regions that modulate the activity of the core, but there are examples of minimal DBRHs that consist of the helicase core only such as eIF4A and *B*. *subtilis* YfmL. For these DBRH other proteins might contribute to binding to the RNA target. An example of this is eIF4A that functions in initiating translation initiation in eukaryotes and is part of a complex (Andreou and Klostermeier, 2013).

In bacteria, DBRHs are associated with ribosomal biogenesis and mRNA degradation through degradosome-like complexes (reviewed in Redder et al., 2015). However, it is unclear how these proteins recognize their authentic substrates or how their activities are regulated *in vivo.* This gene family is highly conserved in bacteria and there is no evidence of horizontal transfer (López-Ramírez et al., 2011). *B. subtilis* has four genes encoding DEAD-box RNA helicases, all belong to the SF2 family: *cshA, cshB, deaD* (formerly known as *ydbR, yqfR* and *yxiN*, respectively), and *yfmL*. The *yfmL* gene appears to have evolved in the Firmicutes, through a duplication from the *cshB* gene, and it only occurs in this phylum (López-Ramírez et al., 2011). YfmL is one of the smallest DEAD helicases, with a C-terminal end of only 41 residues. Phylogenetic analysis shows that the DeaD helicases of Firmicutes form a sister clade with that of CshA, suggesting that they originated from a duplication (López-Ramírez et al., 2011).

The *B. subtilis* DBRH participate in different aspects of RNA metabolism, but they all converge in the processing of rRNA (**Table 1**). They have an important role in ribosome biogenesis, as CshA and YfmL affect 50S biogenesis, and CshB alters 70S biogenesis (Lehnik-Habrink et al., 2013). DeaD/YxiN has been shown *in vitro* to be specific for processing a particular loop present in the 23S rRNA (part of subunit 50S) (Kossen and Uhlenbeck, 1999). The known protein–protein interactions of the different helicases (Lehnik-Habrink et al., 2013) have also revealed an overlap: CshA, CshB, and DeaD interact with PnpA, and of CshA and CshB interact with PykA (Lehnik-Habrink et al., 2013). Single mutants of DBRHs can grow well at 37°C but exhibit growth defects at 18°C, except for DeaD mutants that at 18°C grow as well as wild type (Lehnik-Habrink et al., 2013). The expression of all four genes has been shown to occur during vegetative growth, but only CshA maintains its expression until stationary phase (Lehnik-Habrink et al., 2013). Microarray analysis showed an increased expression for *deaD* and *cshA* during early spore development (Nicolas et al., 2012).

**Table 1:** Summary of DEAD-box RNA helicase functions and epistatic interactions.

DEAD-box RNA helicase	Function	Phenotype of single mutant	Protein interactions	Epistasis/Cross complementation	
DeaD/	Ribosomal RNA	Delayed entry into	Loop 92	CshA:	Negative/Negative
YxiN	processing	sporulation^1^	(23S rRNA)^2^		
		Increased *deaD* and *yfmL* transcripts		CshB:	Negative/Negative
				YfmL:	Negative/Negative
CshA/	Aids in mRNA degradation	Cold-sensitive^3,4^	Ribosomal proteins L1 and L3^4^	CshB:	Positive/Negative
YdbR	Assembly of the 50S	Altered ribosome pattern^4^	RNases J1 and Y^4^		
		Delayed entry into sporulation^1^	PnpA^4^		
		Increased *cshB* transcript			
				YfmL:	Positive/Negative
CshB/	Aids in mRNA degradation	Cold-sensitive^3,4^	Cold shock protein B (CspB)^3^	YfmL:	Positive/Negative
YqfR	Biogenesis of mature 70S ribosomes	Altered ribosome pattern^4^			
		Delayed entry into sporulation^1^ Increased *deaD* transcript			
YfmL	Cold adaptation	Cold-sensitive^4^			
	Assembly of 50S	Altered ribosome pattern^4^			
		Delayed entry into			
		sporulation^1^			
		Increased *deaD, cshA, cshB* transcripts			

*^1^This study, ^2^(Kossen and Uhlenbeck, 1999), ^3^(Hunger et al., 2006), ^4^(Lehnik-Habrink et al., 2013).*

In this work, we explored whether in *B. subtilis* the absence of a phenotype under some conditions in single DBRH deletion mutants (Lehnik-Habrink et al., 2013), could be explained by the redundancy afforded by the conserved helicase SF2 core in the different paralogs. We evaluated if double mutants would exhibit epistatic effects and if there was genetic compensation. Epistatic effects were observed that may result from the participation of the DBRH in the same metabolic pathways, but no cross-complementation was observed suggesting the lack of a functional overlap. We also analyzed chimeras between YfmL, the smallest DBRH in bacteria, and the C-terminal end of CshA, CshB, and DeaD/YxiN to determine if the combination of functional modules would results in hybrids that would complement the mutants or if interference would ensue if the C-terminal end guided recruitment to different substrates. No complementation was observed with any of the chimeras. Instead, these chimeras were lethal on some of the mutants’ backgrounds. We suggest that this negative effect could be the explained from the chimeras interfering in the pathway of ribosome assembly. An additional observation was that all DBRH mutants exhibited a delay in sporulation that was more severe for the Δ*deaD/yxiN*, the first phenotype reported for this mutant. Our results show that the *B. subtilis* DBRHs paralogs are not capable of complementing each other and therefore these duplicated genes cannot buffer one another.

## Materials and Methods

### Strains and Growth Conditions

All *B. subtilis* strains used in this study are listed in **Table 2**. Single mutants GP1051 and GP1083 (Lehnik-Habrink et al., 2013) were modified to change their cassette resistance. The chloramphenicol cassette was exchanged by an erythromycin cassette using plasmid pIC177 (Steinmetz and Richter, 1994), generating strains GOB28 and GOB29.

Strain construction by transformation using chromosomal or plasmid DNA was performed by a conventional two-step procedure (Cutting and Vander Horn, 1990). DNA was isolated by lysing 10 ml pellets from an overnight culture. Lysis was done by adding 2 ml lysis buffer (50 mM EDTA, 0.1M NaCl, pH 7.5), 10 mg lysozyme (USB^®^), and 300 μl sarkosyl (20% w/v), and incubating for 10 min at 37°C. The DNA was then extracted with phenol/chloroform and precipitated with isopropanol. Transformants were selected on semisolid LB plates with selection for the appropriate antibiotics associated with the cassette interrupting the respective genes [kanamycin (10 μg/ml), chloramphenicol (5 μg/ml), tetracycline (20 μg/ml), or erythromycin (1 μg/ml)]. For each transformant, at least two colonies were isolated and disruption of the gene was verified by PCR. To obtain the growth curves, single colonies were inoculated into LB medium, and cultures were grown at 37°C until a Klett absorbance of 100 was reached and 1 ml was inoculated into 30 ml of fresh LB medium liquid and incubated 18°C with agitation. To calculate the relative growth rate (W) the maximum growth rate was normalized using wild type growth value.

### Construction and Evaluation of Overexpression Strains

The *deaD* gene was amplified using PX-*deaD* oligonucleotides (**Supplementary Table S1**), and the PCR product was inserted into the *Bam* HI site of plasmid pX (Kim et al., 1996). The resulting plasmid was transformed into *E. coli* MC106 (RecA^+^), and finally used to transform *B. subtilis* GP1052 strain to generate GOB627.

### Construction of YfmL Core Chimeras and Analysis in wt and Mutant Backgrounds

The complementation of single mutants was analyzed by overexpression of chimeras with a native core region from YfmL and different C-terminal ends. The four DBRH sequences were aligned by Clustal Omega and SWISS-PROT. Conserved Core, variable C-terminal end, and secondary structure was established. The 1 kb sequence corresponding to the YfmL core was amplified (corresponding to amino acid residues 1 to 335, last four residues are GRAG) and a *Spe*I restriction site was inserted at the amino-terminal end. The sequence corresponding to the variable C-terminal of each RNA helicases was amplified, starting at residue 354 (KTG) for *cshA*; 341 (SSG) for *cshB*; 336 (NKG) *deaD*/*yxiN*. The length for each was *cshA*, 477 bp; *cshB*, 297 bp; and *deaD*, 435 bp and *Bam*HI sequence was inserted within the reverse oligonucleotide (see oligonucleotides used in **Supplementary Table S1**). The corresponding *yfmL* core and variable C-terminal segments were fused by double-joint PCR (Yu et al., 2004) generating *yfmL-cshA*, *yfmL-cshB*, and *yfmL-deaD* fusions. The chimeras were inserted into the pX plasmid in the *Spe*I and *Bam*HI restriction sites. The constructs were sequenced to verify accuracy. *B. subtilis* wt and single mutants were transformed by a conventional two-step procedure (**Table 2**). The overnight cultures were serially diluted by factors of 10, spotted on LB with and without xylose, and incubated at 37°C, 18°C and 10°C. Triplicate platings and biological replicas were done [photographs of the plates at 37°C (**Supplementary Figure S1**) and 18°C].

### Epistasis Determination

Epistasis between paralogous genes (double mutants) was evaluated as in DeLuna et al. (2008). The mean of maximum growth rate of double mutant kinetics was normalized in reference to wild type, and considered relative growth rate (W). The difference between the fitness of the double deletion strain and the additive expectation yields is the epistasis degree £ = Wx′x″ – Wx′ Wx″ (DeLuna et al., 2008). As for nomenclature on epistasis, we adopted the suggestion of Phillips (2008) and referred simply to positive epistasis when the phenotype is higher than expected (growth, in this case) or negative epistasis if the phenotype is lower than expected.

**Table 2:** *B. subtilis* strains used in this study.

Strain	Genotype	Reference
168	*trpC2*	Lehnik-Habrink et al., 2013
GP1051	*trpC2 ΔcshB::cat*	Lehnik-Habrink et al., 2013
GP1052	*trpC2 ΔdeaD::tet*	Lehnik-Habrink et al., 2013
GP1053	*trpC2 ΔyfmL::mls*	Lehnik-Habrink et al., 2013
GP1083	*trpC2 ΔcshA::cat*	Lehnik-Habrink et al., 2013
GOB628	*trpC2 ΔcshB::mls*	This study
GOB629	*trpC2 ΔcshA::mls*	This study
GOB661	*trpC2 ΔcshA::cat ΔcshB::mls*	This study
GOB662	*trpC2 ΔcshA::cat ΔdeaD::tet*	This study
GOB663	*trpC2 ΔcshA::cat ΔyfmL::mls*	This study
GOB664	*trpC2 ΔcshB::cat ΔdeaD::tet*	This study
GOB665	*trpC2 ΔcshB::cat ΔyfmL::mls*	This study
GOB666	*trpC2 ΔdeaD::tet ΔyfmL::mls*	This study
GP1084	*trpC2 ΔcshA::cat lacA::cshA aphA3*	Lehnik-Habrink et al., 2013
GP1086	*trpC2 ΔcshB::cat lacA::cshB aphA3*	Lehnik-Habrink et al., 2013
GP1087	*trpC2 ΔyfmL::mls lacA::yfmL aphA3*	Lehnik-Habrink et al., 2013
GOB627	*trpC2 ΔdeaD::tet amyE::deaD cat*	This study
GOB630	*trpC2 ΔcshA::cat lacA::cshB aphA3*	This study
GOB631	*trpC2 ΔcshA::mls amyE::deaD cat*	This study
GOB632	*trpC2 ΔcshA::cat lacA::yfmL aphA3*	This study
GOB633	*trpC2 ΔcshB::cat lacA::cshA aphA3*	This study
GOB634	*trpC2 ΔcshB::mls amyE::deaD cat*	This study
GOB635	*trpC2 ΔcshB::cat lacA::yfmL aphA3*	This study
GOB636	*trpC2 ΔdeaD::tet lacA::cshA aphA3*	This study
GOB637	*trpC2 ΔdeaD::tet lacA::cshB aphA3*	This study
GOB638	*trpC2 ΔdeaD::tet lacA::yfmL aphA3*	This study
GOB639	*trpC2 ΔyfmL::mls lacA::cshA aphA3*	This study
GOB640	*trpC2 ΔyfmL::mls lacA::cshB aphA3*	This study
GOB641	*trpC2 ΔyfmL::mls lacA::deaD aphA3*	This study
GOB642	*trpC2 lacA::cshA aphA3*	This study
GOB643	*trpC2 lacA::cshB aphA3*	This study
GOB644	*trpC2 amyE::deaD cat*	This study
GOB645	*trpC2 lacA::yfmL aphA3*	This study
GOB667	*trpC2 amyE::yfmL-cshA Cm*	This study
GOB755	*trpC2 amyE::yfmL-cshB Cm*	This study
GOB763	*trpC2 amyE::yfmL-deaD Cm*	This study
GOB668	*trpC2 ΔcshA::mls amyE::yfmL-cshA Cm*	This study
GOB754	*trpC2 ΔcshA::mls amyE::yfmL-cshB Cm*	This study
GOB766	*trpC2 ΔcshB::mls amyE::pxylA Cm*	This study
GOB757	*trpC2 ΔcshB::mls amyE::yfmL-cshA Cm*	This study
GOB758	*trpC2 ΔcshB::mls amyE::yfmL-cshB Cm*	This study
GOB759	*trpC2 ΔcshB::mls amyE::yfmL-deaD Cm*	This study
GOB765	*trpC2 ΔdeaD::Tet amyE::pxylA Cm*	This study
GOB768	*trpC2 ΔdeaD::tet amyE::yfmL-cshA Cm*	This study
GOB764	*trpC2 ΔdeaD::tet amyE::yfmL-cshB Cm*	This study
GOB760	*trpC2 ΔdeaD::tet amyE::yfmL-deaD Cm*	This study
GOB767	*trpC2 ΔyfmL::mls amyE::pxylA Cm*	This study
GOB761	*trpC2 ΔyfmL::mls amyE::yfmL-cshA Cm*	This study
GOB756	*trpC2 ΔyfmL::mls amyE::yfmL-cshB Cm*	This study
GOB762	*trpC2 ΔyfmL::mls amyE::yfmL-deaD Cm*	This study
GOB667	*trpC2 amyE::yfmL-cshA Cm*	This study
GOB755	*trpC2 amyE::yfmL-cshB Cm*	This study
GOB763	*trpC2 amyE::yfmL-deaD Cm*	This study
GOB668	*trpC2 ΔcshA::mls amyE::yfmL-cshA Cm*	This study
GOB754	*trpC2 ΔcshA::mls amyE::yfmL-cshB Cm*	This study
GOB754	*trpC2 ΔcshA::mls amyE::yfmL-deaD cm*	This study
GOB766	*trpC2 ΔcshB::mls amyE::pxylA Cm*	This study
GOB757	*trpC2 ΔcshB::mls amyE::yfmL-cshA Cm*	This study
GOB758	*trpC2 ΔcshB::mls amyE::yfmL-cshB Cm*	This study
GOB759	*trpC2 ΔcshB::mls amyE::yfmL-deaD Cm*	This study
GOB765	*trpC2 ΔdeaD::Tet amyE::pxylA Cm*	This study
GOB768	*trpC2 ΔdeaD::tet amyE::yfmL-cshA Cm*	This study
GOB764	*trpC2 ΔdeaD::tet amyE::yfmL-cshB Cm*	This study
GOB760	*trpC2 ΔdeaD::tet amyE::yfmL-deaD Cm*	This study
GOB767	*trpC2 ΔyfmL::mls amyE::pxylA Cm*	This study
GOB761	*trpC2 ΔyfmL::mls amyE::yfmL-cshA Cm*	This study
GOB756	*trpC2 ΔyfmL::mls amyE::yfmL-cshB Cm*	This study
GOB762	*trpC2 ΔyfmL::mls amyE::yfmL-deaD Cm*	This study
GP1010	*trpC2 cshA*-FLAG *spc*	Lehnik-Habrink et al., 2013
GP1011	*trpC2 cshB*-FLAG *spc*	Lehnik-Habrink et al., 2013
GP1066	*trpC2 yfmL*-FLAG *spc*	Lehnik-Habrink et al., 2013
GP1068	*trpC2 deaD*-FLAG *spc*	Lehnik-Habrink et al., 2013
GOB946	*trpC2 ΔcshA::cat cshB*-FLAG *spc*	This study
GOB947	*trpC2 ΔcshA::cat deaD*-FLAG *spc*	This study
GOB948	*trpC2 ΔcshA::cat yfmL*-FLAG *spc*	This study
GOB949	*trpC2 ΔcshB::cat cshA*-FLAG *spc*	This study
GOB950	*trpC2 ΔcshB::cat deaD*-FLAG *spc*	This study
GOB951	*trpC2 ΔcshB::cat yfmL*-FLAG *spc*	This study
GOB952	*trpC2 ΔdeaD::tet cshA*-FLAG *spc*	This study
GOB953	*trpC2 ΔdeaD::tet cshB*-FLAG *spc*	This study
GOB954	*trpC2 ΔdeaD::tet yfmL*-FLAG *spc*	This study
GOB955	*trpC2 ΔyfmL::mls cshA*-FLAG *spc*	This study
GOB956	*trpC2 ΔyfmL::mls cshB*-FLAG *spc*	This study
GOB957	*trpC2 ΔyfmL::mls deaD*-FLAG *spc*	This study

### Bacterial Growth Fixed to Logistic Model

This model describes the self-limiting growth of a biological population (Verhulst, 1845). Experimental data was used to calculate maximum growth rate (ri) and carrying capacity (Ki), through a non-linear regression in R software (R Development Core Team, 2008) and Gauss-Newton algorithm. The individual and general correlation coefficient (*r*^2^) was calculated. The double mutant growth was analyzed in function to growth rate, using ANOVA and Tukey’s test with a confidence level of 0.95.

### qPCR Expression Analysis

To measure each helicase expression in single mutant backgrounds, total RNA was isolated using TRIZOL^®^ (Ambion), as described by the manufacturer. RNA was cleaned up using the PureLink^®^ RNA Mini Kit (Invitrogen), according to the manufacturer’s instructions. The cDNA was synthesized following the SuperScript III Reverse transcriptase protocol (Invitrogen), with variations in the “First-Strand cDNA Synthesis.” The reaction contained 1 μl of a cocktail of gene-specific primers at 2 pmol (*sigA*-R, *gyrA*-R, *cshA*-R, *cshB*-R, *deaD*-R, *yfmL*-R; sequences are listed in **Supplementary Table S1**) (Liles et al., 2004), 10 pg to 5 μg of total RNA, 2 μl 10 mM dNTP mix, and sterile distilled water to 13 μl. Reverse primers were designed to have the anchor sequence MYT4 to ensure the amplification only of the desired transcripts (Gadkar and Filion, 2013). The rest of the synthesis was as suggested by the supplier. Quantification was performed in a StepOne^TM^ instrument (Applied Biosystems), using the EXPRESS SYB^®^ kit (Invitrogen), forward primers *sigA*-F, *gyrA*-F, *cshA*-F, *cshB*-F, *deaD*-F, *yfmL*-F and the Acopl as reverse primer in each reaction. The amplification protocol used for PCR was 95°C (3 min), 35 cycles of 95°C (10 s) and 60°C (30 s). The quantification of relative expression was performed with 2^−ΔΔCT^ method, as described in the Applied Biosystems Bulletin No. 2 (P/N 4303859). The endogenous controls were *sigA* and *gyrA* genes in the wild type background were used to calculate relative expression. Arbitrarily, two levels of increased expression were established; the first between 2 and 5 relative fold ratio, and the second one up to fivefold.

### Protein Expression Analysis

To monitor the expression patterns of the DEAD-box RNA helicases, we used strains expressing the helicases labeled with a C-terminal triple FLAG tag. The respective strains for CshA, CshB, DeaD, and YfmL were available (Lehnik-Habrink et al., 2013). Single mutants were transformed with chromosomal DNA thus replacing the respective genes with a Flag- tagged construct.

LB media cultures were grown at 18 and 37°C until the exponential phase. Cells were harvested by centrifugation (950 × *g*, 10 min, 25°C) and the pellet washed (with 5 mL Tris 50 mM, pH 8.4, KCl 1 M) and lysed with a buffer (Tris 50 mM, pH 8.4, KCl 1 M) containing 1X protease inhibitor (Thermo Fisher), 1X DNase (Sigma-Aldrich), 50 μg/ml lysozyme (USB^®^) and centrifuged (12,000 × *g* 15 min at 4°C). The aqueous phase was separated (Cutting and Vander Horn, 1990). The protein quantification was determinated using Bio-Rad’s Bradford reagent.

For Western blot analysis, proteins were separated on a 12% polyacrylamide gel and transferred onto nitrocellulose membrane (Bio-Rad) by electroblotting. Rabbit anti-FLAG polyclonal antibodies (Sigma-Aldrich; 1X) served as primary antibody. The antibodies were visualized by using 100 μl Clarity Western ECL Substrate (Peroxide solution) and 100 μl Clarity Western ECL Substrate (Luminol/enhancer solution) from Bio-Rad (Lehnik-Habrink et al., 2010).

### Determination of Sporulation Kinetics

Sporulation was determined for the four single DEAD-box RNA helicase mutants (GP1050, GP1052, GP1053, and GP1083). For sporulation assays, the cells were pre-cultured on LB-agar plates at 28°C overnight and then recovered with 2 ml of Difco sporulation medium (DSM); Bacto Nutrient Broth, 8% (w/v); KCl, 0.1% (w/v); MgSO_4_, 0.012%; and 1 ml of the following salt stocks: Ca(NO_3_)_2_, 1 M; MnCl_2_, 0.01 M and FeSO_4_ 1 mM. Klett flasks containing 30 ml of fresh DSM medium were inoculated to a Klett absorbance of 5–9. Inoculated flasks were kept under agitation at 37°C. Klett absorbance was monitored each 30 min until cultures were clearly in the stationary phase. The beginning of the sporulation process was determined by plotting the Klett absorbance against time, with the initiation of sporulation (*t*_0_) matching the starting of the stationary phase. Samples were collected as triplicates at 7, 8, 9, 10, 12, and 30 h after *t*_0_. Glycerol was added to a 20% final concentration and the samples were immediately frozen in liquid nitrogen and kept until tested for sporulation. Spores percentages were obtained by titering cells before and after heat treatment at 85°C for 20 min, plating in triplicate on LB-agar. The results are the mean of three independent experiments made by triplicates. T-student analysis was performed on data of each mutant strain in relationship to the wild type at each time.

## Results

### Double Mutant Phenotypes and Epistatic Relationship Between DEAD-Box RNA Helicases Genes

We analyzed double mutants to determine if there were buffering interactions between DBRH genes. We wondered if the lack of a growth defect at 37°C in DBRH mutants could be the result of redundancy. The growth of the double mutants at 37°C was observed to be similar to that of the single mutants. Only the Δ*cshB* Δ*deaD* double mutant exhibited decreased growth rate (**Supplementary Figure S2** and **Supplementary Table S2**). This result suggested that the lack of a phenotype for growth at 37°C was not explained by a buffering effect among helicases.

**FIGURE 2:**
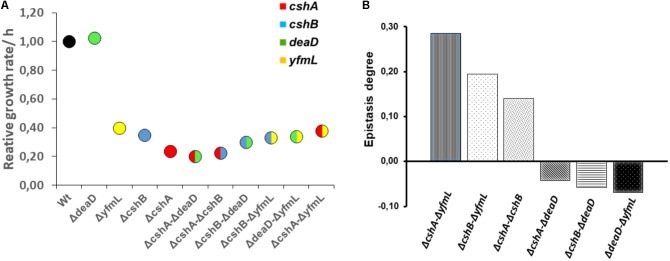
Growth rates of single and double mutants and differential epistasis between RNA helicase genes. **(A)** Circles are means of triplicate incubates samples. Growth was evaluated at 18°C and the data fixed to a logistic model was normalized with wild type value. Data was analyzed on R using ANOVA and Tukey test at 0.95 confidence level. **(B)** Epistasis degree at 18°C is expressed as the difference between relative maximum growth rate of double mutant (Wx’x”) and relative maximum growth rate expected in non-epistatic interaction (Wx’ ^∗^ Wx”): £ = Wx’x” – Wx’ Wx” (DeLuna et al., 2008). Values above zero mean positive epistasis while values below zero suggest negative epistasis.

To determine the degree of epistasis between the DBRH, we calculated the relative growth rate of single and double mutant strains at 37°C, where growth is only slightly affected, as well as at 18°C, since at this temperature growth is noticeably affected for the *cshA*, *cshB* and *yfmL* mutants (Lehnik-Habrink et al., 2013). The growth rate of double mutants at 37°C was not greatly affected, and on the contrary, the double mutants Δ*cshB*/Δ*deaD* grew better than the corresponding single mutants (**Supplementary Figure S2**). The growth rate at 18°C for the double mutants was in most cases the same as that of single mutants (**Table 3**). An exception was the Δ*cshA ΔyfmL* strain that grew better than the *cshA* single mutant strain (**Table 3** and **Figure 2A**). Epistasis between two mutations is defined as the degree to which the effect of both mutations together differs from the sum of the consequences of the single mutations (DeLuna et al., 2008). Positive epistasis was most noticeable for Δ*cshA*Δ*yfmL* and less pronounced for the Δ*cshA*Δ*cshB* and Δ*cshB*Δ*yfmL* double mutants, since the observed growth of the double mutant was greater than that of the calculated added growth (**Figure 2B**). On the contrary, epistatic values for Δ*deaD* combinations were slightly negative (very close to zero) suggesting little or no epistatic relationships with other DEAD-box RNA helicase genes.

**Table 3:** Differences of maximum and relative growth rate of simple and double DEAD-box RNA helicase mutants at 18°C.

	Growth rate (r/h) ± SD	Relative growth (W)^∗^
*Wild type*	0.390 ± 0.007 a	1.00
*ΔcshA*	0.092 ± 0.001 e	0.24
*ΔcshB*	0.136 ± 0.005 bcd	0.35
*ΔdeaD*	0.400 ± 0.014 a	1.03
*ΔyfmL*	0.155 ± 0.010 b	0.40
*ΔcshA::cat ΔcshB::mls*	0.087 ± 0.001 e	0.22
*ΔcshA::cat ΔdeaD::tet*	0.078 ± 0.005 e	0.20
*ΔcshA::cat ΔyfmL::mls*	0.148 ± 0.004 bc	0.38
*ΔcshB::cat ΔdeaD::tet*	0.117 ± 0.008 d	0.30
*ΔcshB::cat ΔyfmL::mls*	0.130 ± 0.005 cd	0.33
*ΔdeaD::tet ΔyfmL::mls*	0.132 ± 0.005 cd	0.34

*Mean and SD, standard deviation of triplicate samples. Comparative method Tukey HSD, 0.95 confidence level, differences in letters represent significance differences. ^∗^Relative growth (based on a logistic model, Verhulst, 1845).*

### Expression of DEAD-Box RNA Helicases in Different Mutants’ Background Do Not Exhibit a Compensation Pattern

Given that one of the functions of RNA helicases, particularly CshA, is related to RNA degradation through the RNA degradosome, it was possible that in its absence an increased expression of other DBRHs could compensate for the lack of CshA and thus mask the effect of the mutant. To determine to what extent a mutation in a given RNA helicase affected transcription or protein expression of the other DEAD-box RNA helicase genes, we quantified the mRNA of each of the RNA helicase genes in the wild type and in individual mutants’ backgrounds and well as protein expression through assays of FLAG fusions.

We evaluated expression with cultures grown at low temperature (18°C), since the mutant growth phenotypes are best revealed at this temperature. For qPCR *sigA* mRNA was used as the internal control, though other experiments were also carried out with *gyrA* mRNA as an internal control, with similar results (data not shown).

We observed several changes in RNA expression, consistent with RNA helicases participating in RNA metabolism. In the Δ*cshA* strain the main effect observed was on the *cshB* gene, exhibiting the highest genetic expression increase However, this effect was not reciprocal as, in the absence of *cshB*, the expression of *cshA* gene did not increase noticeably (**Supplementary Figure S3** and **Supplementary Table S3**). In the Δ*cshB* mutant, the *deaD* and *yfmL* helicase genes exhibited increased mRNA expression though the main gene affected was *deaD*. When expression was analyzed in the Δ*deaD* mutant, *cshA* and *yfmL* were up-regulated. In the Δ*yfmL* background, all helicase genes were affected with the strongest effect on *deaD*. However, *yfmL* expression did not increase notably in the absence of any other DEAD-box RNA helicase gene (**Supplementary Figure S3**). In summary, although we observed that the loss of each helicase gene modified the expression of the other genes, in no case was a pattern observed that could indicate a compensation effect between particular pairs of DBRHs. We therefore set up to determine if the increase in some mRNA transcriptional level impacted on the expression at the protein level. We carried out Western blot analysis of the mutants carrying C terminal FLAG fusions to each of the RNA helicases. The increased expression observed at mRNA level did not correlate with increased protein expression. It is possible that the mRNA degradation pathway is affected in the mutants and this causes the observed mRNA levels perturbation. In no case was protein expression of a helicase increased in the absence of another one. On the contrary, while CshA-FLAG exhibited no growth phenotype in wild type strain, when introduced into the DBRH mutants’ background caused drastic growth reduction and its expression was greatly reduced; similarly, diminished expression of YfmL-FLAG was observed in the Δ*deaD* background (**Supplementary Figure S4**). In summary, the observed de-regulation of expression did not seem to be meaningful in terms of a genetic compensating mechanism. We therefore set up to directly determine whether any one helicase could substitute for another one.

### Lack of Cross-Complementation Between DEAD-Box RNA Helicases, Confirms the Lack of Compensatory Effects

We evaluated if the different helicases could complement each others function by expression of individual DBRH genes using an inducible promoter. The xylose inducible promoter allowed us to maintain gene expression during growth, and particularly after logarithmic phase when only *cshA* is expressed (Lehnik-Habrink et al., 2013). By inducing expression of the DEAD-box helicase genes, it was possible to evaluate whether overexpression of individual DBRHs interfered with the function of another one, which could be detected as an effect on growth, and if cross-complementation by functional redundancy occurred.

We started by testing the system with self-complementation and evaluating different inducer concentrations. The maximum self-complementation effect was obtained with 1% xylose (data not shown), and this concentration was therefore chosen for the complementation experiments. When DEAD-Box RNA helicase genes were induced in the wild type background, the dosage effect was minimal on the growth phenotype, and no detrimental effect was detected (**Supplementary Figure S5**). Growth rate (r/h) increased substantially for the self-complemented mutants upon addition of xylose, bringing them close to the wild type growth (0.27): CshA (0.09–0.23), CshB (0.17–0.25) and YfmL (0.18–0.23), and no change was observed for DeaD (0.26–0.27). This is consistent with the results from Lehnik-Habrink et al. (2013); this data is shown in **Supplementary Table S4** and **Supplementary Figure S6**. At the stationary phase, all mutants had reached a similar cell density.

**FIGURE 3:**
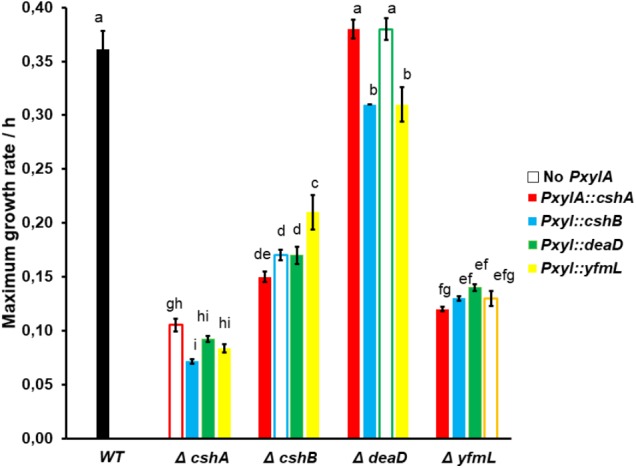
Cross complementation is not observed in single mutants when the different RNA helicase genes are overexpressed. Three samples were grown at 18°C with xylose 1% as the promoter inducer. Growth was monitored until the stationary phase, then maximum growth rate was calculated. The black bar represents the wild type, and the open bar represents the mean single mutant without inducible promoter. ANOVA and Tukey test was applied at 0.95 confidence level, differences in letters represent significant differences.

Regarding cross-complementation analysis, expression in the different mutant backgrounds of the different DEAD-box RNA-helicase genes driven by the pXylA promoter did not result in the recovery of wild type growth for any of the mutants (although for the *deaD* mutant this cannot be determined since the mutant grows as well as the wild type strain) (**Supplementary Table S5**). In the Δ*cshA* background, the overexpression of any other DEAD-box RNA helicase gene had a small but reproducible negative effect on growth. In contrast, in the Δ*cshB* background, overexpression of *yfmL* had a positive effect on growth, although the wild-type growth rate was not reached. Interestingly, an effect was observed even in the in the absence of xylose, suggesting that even a weak expression can affect growth (**Supplementary Table S5**). It is known that the *xylA* promoter is not entirely tight and that some transcript is produced even without an inducer (Kim et al., 1996). In a Δ*deaD* mutant, overexpression of either *cshB* or *yfmL* induced a small but statistically significant reduction in growth. Similarly, overexpressing *cshA* in a Δ*yfmL* mutant background caused a slight decrease in growth, while overexpression of *deaD* and *cshB* improved the Δ*yfmL* phenotype slightly without inducer (**Figure 3**). In summary, these data suggest that the different *B. subtilis* DEAD-box RNA helicases cannot substitute for each other and that this is not a consequence of having evolved different patterns of expression, but due to a lack of overlap of functions in the different ancestral duplicates. Since the full DBRHs cannot substitute one another, we wonder if the conserved SF2 core of one of them, linked to the different C-terminal ends would complement the mutants.

### Chimeras Between the YfmL Core and the C-Terminal End of DBRH Result in Dramatic Growth Phenotypes Consistent With Genetic Interference

**FIGURE 4:**
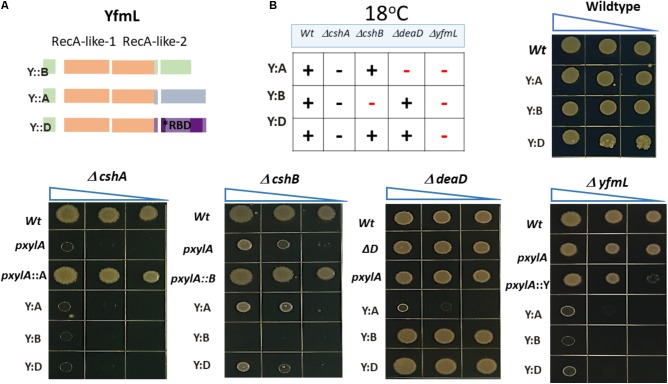
*In vivo* functional analysis of chimeric helicases. **(A)** The YfmL core was used to construct chimeras with the C- terminal end of *cshA*, *cshB* and *dead*. **(B)** The expression of chimeric helicases was driven by the xylose inductor and growth was tested in the wild type and each mutant background. Strains were grown and dilutions were spotted onto LB-agar and incubated at 37°C or 18°C. (+) means optimal growth, (–) black symbol means low growth, (–) red symbol mean negative effect.

DBRH have been described as modular proteins where the N-terminal SF2 core possesses the catalytic ATPase and helicase, and the C-terminal domain determines substrate election (Kossen et al., 2002). We chose YfmL as the helicase domain to construct chimeras, since it is the smallest DBRH, consisting only of this domain (**Figure 1**). Constructions were generated where the C-terminal end of each of the helicases was linked to the YfmL core (**Figure 4**). We expected that the conserved SF2 core of YfmL, once hooked to the different C-terminal ends, would be able to complement the different DBRH mutants. The C-terminal end of all have a calculated isoelectric point in the range of 9.5 and 10 and are rich in Arg and Lys residues. (The complete sequence of the different helicases is shown on **Supplementary Figure S7**, with a table showing the percent content of charged amino acids.) Only DeaD/YxiN is known to have a domain that confers specificity to the 23S rRNA (Wang et al., 2006). These constructions were introduced into the wt background as well as into the different mutants. In the wt strain no growth differences could be observed, either at 37°C or 18°C, suggesting that the chimeras did not have a negative effect. In the single mutants no negative effect on growth was observed at 37°C. However, at 18°C where single mutants of Δ*cshA* and Δ*cshB* exhibit deficient growth, we expected to observe improved growth if there was complementation, but this was not the case for any of them. Obviously the SF2 core of YfmL was not able to replace that of CshA, CshB or DeaD/YxiN. Instead, we were surprised by the strong negative effect that the YfmL’CshB chimera had on the Δ*cshB* growth. Similarly, YfmL’CshA impaired growth of Δ*deaD* **Figure 4** summarizes these results. It is interesting that all of the chimeras impaired Δ*yfmL* growth. It is also interesting the negative effect of YfmL’CshA chimera in a Δ*deaD* background, for which no phenotype associated to rRNA biogenesis had been observed.

### Further Evidence of Subfunctionalization: Sporulation Delay of DBRH Mutants, Particularly Severe for a *deaD* Mutant

**FIGURE 5:**
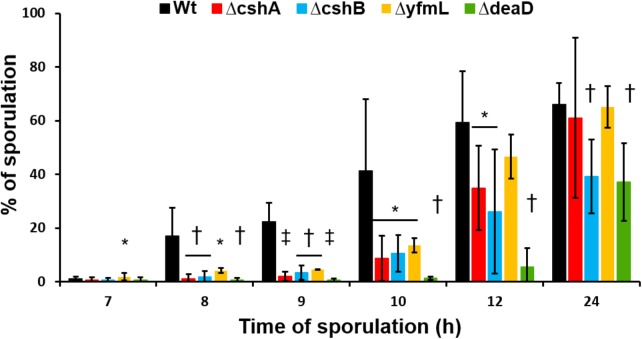
Sporulation kinetics of RNA-helicase mutant strains. Sporulation of mutant strains and wild type was induced in DSM at 37°C. Samples were taken at 7, 8, 9, 10, 12, and 24 h after the beginning of stationary growth phase. Values are the mean of three independent experiments. ^∗^*P* < 0.5, ^†^*P* < 0.1, and ^‡^*P* > 0.01 when compared with wild type at the same sporulation time.

Since the *B. subtilis* DBRH perform specific roles in ribosome biogenesis and RNA degradation (Ando and Nakamura, 2006; Kossen et al., 2002; Lehnik-Habrink et al., 2013) and, as we show in this study, they do not complement each other it is intriguing that none of them is essential for growth. DeaD is particularly intriguing, since even a specific target on the 23S rRNA was described (Wang et al., 2006), and it is the one mutant for which no defect had been observed. The *deaD* mutant does not exhibit a growth phenotype even at low temperature. We examined other growth conditions, such as low pH, different temperatures, and osmotic conditions, and found no differences from wild type (data not shown). We examined the sporulation phenotype of the mutants since DBRH gene expression increases at time 2 of sporulation initiation (Nicolas et al., 2012). Each single mutant was grown in DSM, a medium routinely used to induce sporulation in *B. subtilis* (Cutting and Vander Horn, 1990). We determined the sporulation percentage of wild type and each mutant at intervals of 7, 8, 9, 10, 12, and 24 h after the onset of sporulation (t_0_) (**Figure 5**). At T_7_, none of the strains had yet sporulated, consistent with the previously described timing for *B. subtilis*, as the heat-resistance in spores is acquired by time t_8_. For the wild type strain, sporulation was above 10% at T_8_ and increased with the time until it reached ∼66% at T_24_. Interestingly, none of the DEAD-box helicase mutants achieved percentages of sporulation above 10% until T_12_. Despite the delay in the entry into sporulation, Δ*cshA* and Δ*yfmL* finally reached the same sporulation levels as the wild type at T_24_. Student *t*-test showed significant differences of the mutants Δ*cshB* and Δ*deaD* in comparison with the wild type at T_24_, suggesting that these mutants are the most affected in the sporulation process. Our results are compatible with data showing that the four DBRHs’ genes have differential expression during sporulation^1^ (Michna et al., 2016), and suggests their involvement in sporulation initiation or development. The effect on sporulation initiation could be the result of the participation of the DBRH in mRNA degradation of specific mRNAs or a facet of ribosome assembly associated with translation of a gene required for sporulation initiation. To our knowledge, this is the first report describing an altered phenotype for a Δ*deaD* mutant. The particularly strong phenotype of Δ*deaD* mutant constitutes yet more evidence of the subfunctionalization of DBRHs.

## Discussion

An important question in evolutionary biology relates to the role of gene duplication and epistasis in the plasticity of a genome. The four DBRH in *B. subtilis* have a common origin and a highly conserved SF2 core (**Figure 1**). Lehnik-Habrink et al. (2013) observed differences in expression and in specific phenotypes related to ribosomal biogenesis that suggested independent functions for these helicases, suggesting subfunctionalization. The four *B. subtilis* DBRH are thus a good model to evaluate to what extent they have maintained overlapping functions and if they exhibit epistatic interactions.

If there was genetic buffering by a paralog, we would expect a single mutation to have no phenotype (and this is indeed the case at 37°C) but double mutants to exhibit a growth defect if DBRHs complemented each other. We expected a Δ*cshA* Δ*cshB* double mutant to have a negative epistatic effect, as they both participate not just in rRNA biogenesis but also in mRNA degradation. However, none of the DBRHs double mutants exhibited a defective growth at 37°C, suggesting that the absence of a phenotype for growth was not caused by buffering activity through another helicase. Only at 18°C was epistasis revealed for some of the RNA helicases. Contrary to our expectations, double mutants did not grow less than single mutants. Only the Δ*deaD* mutant exhibited a slight negative epistasis in all combinations. In fact, positive epistasis was observed for several double mutants. The most noticeable was that of Δ*cshA* whose impaired growth was improved when combined with Δ*yfmL* and for the Δ*cshA* Δ*cshB* double mutant.

A direct measure of redundancy is the cross expression of the different DBRH genes in the different mutant backgrounds. If there were redundancy, the expected outcome would be the correction of growth to the wild type level, or at least amelioration of the defective growth. We observed that overexpression of each of the four DEAD-box RNA helicases in the wild type background had only a minor effect, suggesting that *B. subtilis* can deal very well with fluctuation in genetic expression of DBRHs. In the different mutant’s background, cross expression did not reestablish wild type growth and, in some cases, resulted in less growth. This result contrasts with the results of crossed complementation observed in *E. coli* where overexpression of the DBRH RhlE can complement the Δ*deaD* cold-sensitive phenotype (Awano et al., 2007). Also, overexpression of CsdA could rescue defects caused by deletion of *srmB* (Charollais et al., 2004).

Another example of conserved functional overlap between paralogs has been reported by Noda-Garcia et al. (2017). They showed that in *B. subtilis* a dramatic negative fitness effect is observed upon promoter swapping of paralogs genes *rocG* and *gudB*, encoding enzymes for the deamination of glutamate. The divergence of their enzymatic regulation leads to detrimental metabolic unbalance upon expression under diverse growth conditions, remarking the importance of paralog-specific regulation. We suggest that, in contrast to paralogs that conserve the same function, such as GudB and RocG, the *B. subtilis* DBRHs have evolved toward functional specialization in which minimal or no overlap of function has remained.

Another aspect of interests in the evolution of SF2 RNA helicases is whether the conserved SF2 core is actually modular regarding the C-terminal end. The C-terminal end, enriched in positively charged residues, is not believed to work through specific motifs, but to bind diverse substrates. The divergent evolution of the *B*. *subtilis* paralogous genes is evident from differences in their carboxy terminal regions, that are predicted to determine substrate specificity. We thought that maybe by shuffling the C-terminal ends we could uncover functional conservation of the core. One way to test this was to evaluate whether the conserved RecA-like core was interchangeable, and in general to explore whether the *B. subtilis* DBRH genes could work as modular proteins. We constructed chimeras using as the helicase core the smallest known bacterial DBRH, YfmL, and fused to it the C-terminal end of each of the three other DBRH. Banroques et al. (2011) tested diverse chimeras from yeast DBRH that led him to propose the minimal functional helicase domain (35 residues after domain VI, see **Figure 1**) and showed, that *in vivo*, chimeras of the helicase and C-terminal domains of Dbp1 and Ded1 could be interchanged (these had, 83% sequence identity). For bacteria, DBRH chimeras have been used to evaluate protein–protein interactions. Lehnik-Habrink et al. (2010) tested a CshB’CshA chimera and determined that the C-terminal end of CshA was sufficient to mediate the interaction with RNase Y. In another study, the C-terminal domain of DeaD/YxiN was fused to the N-terminal *of E. coli* SrmB. The chimera maintained helicase properties of SrmB and the substrate specificity of DeaD/YxiN (Karginov et al., 2005). In contrast, our results showed that none of the chimeras complemented their respective mutants. The SF2 core of YfmL cannot substitute for the function of the other DBRHs SF2 cores. On the contrary, the chimeras were lethal in some DBRH mutant backgrounds. Since the chimeras impaired growth only at 18°C and not at 37°C, the lethal effect seems not to be unspecific but related to RNA metabolism (the kinetics of RNA folding are highly dependent on chaperones at 18°C). Interestingly, the chimeras did not act as dominant negative mutants, since in the presence of the wild type copy of the gene no negative effect was observed. This suggests that the chimeras do not interfere with the dynamics of the wt DBRH. The fact that all the chimeras impede the growth of Δ*yfmL* is very interesting. Since all DBRH participate in rRNA assembly possibly intervening in sequential steps, the chimeras might cause rRNA population to become kinetically trapped in misfolded intermediates that can’t be resolved in the mutants. Since the expression of the chimeras in the Δ*yfmL* background is lethal we cannot test this hypothesis through the analysis of rRNA. In the future, pull down experiments could be done to define the binding substrates of the chimeras.

We also report that all DBRH mutants affect sporulation initiation, with the strongest effect observed for Δ*deaD*. However, at this moment we do not know what causes this phenotypes, as these could be associated to DBRH’s deficiencies in ribosome biogenesis, participation in the degradosome-like complex or translation initiation (Redder et al., 2015). With more than 150 genes known to be involved in sporulation, a single gene or the simultaneous effect on several of them could have this phenotype. Recently, DBRH genes were identified in a screen for a phenotype of delayed sporulation using a transposon library (Meeske et al., 2016). Among the genes found were YfmL (381.5-fold difference) and CshB (238-fold difference); a small effect on DeaD was observed (1.3-fold) while CshA was not present. Other genes involved in RNA metabolism were also uncovered in the screen. These results suggest that alterations in the intrincated metabolism of RNA can affect the function of many genes associated to different processes, including sporulation. Finally, Lehnik-Habrink et al. (2013) used a microarray to evaluate the effect of a CshA mutant on the expression of the *B. subtilis* genes. Only five genes related to sporulation were found to exhibit a small effect, including less than a twofold difference in expression compared to wt expression: *cotD* (0.8), *spoIIB* (0.8), *cotSA* (0.98), *spoVAF* (1.4), and *spoVFE* (1.3). It is not possible to tell whether the modification of one of these genes, or the simultaneous defect of more than one, is responsible for the delayed sporulation effect observed in the CshA mutant. These results suggest that alterations in the intrincated metabolism of RNA can affect the function of many genes associated to different processes, including sporulation.

Our results show that dispensability at 37°C is not explained by considering the different DBRHs paralogs as backup genes, and rather that at this temperature RNA unfolding dynamics make these enzymes non-essential. Since DBRHs do not perform the same tasks but work on consecutive interconnected pathways, particularly in rRNA biogenesis, each others functions are interdependent, thus explaining in part their epistatic interactions. We suggest that the expansion of the DBRH optimized an essential function in bacteria, ribosome processing, and assembly, as well as other aspects of RNA metabolism. We propose that for the four *B. subtilis* DBRH to work on the assembly line for the production of the 70S rRNA, no competing function could exist and that, therefore, these helicases evolved avoiding redundancy conflicts. Given the conflict encountered by the presence of a modified SF2 core linked to a different C-terminal end (the YfmL chimera), we suggest that the duplication route required that changes in the C-terminal occurred before those in the helicase domain, so as not to compete with the paralog. Optimal coordination of both domains would then slowly take place through coevolution. The articulation required for the function of the SF2 and its C-terminal end constrains the possibilities for modularity. The protein biochemistry of the individual proteins must be further investigated to better understand their specialization.

In summary, our study led us to conclude that there is little functional overlap among the *B. subtilis* DBRHs. The fact that none of the double mutants is lethal could be explained by the robust cell machinery involved in RNA metabolism and not by a buffering activity among them. We suggest that the epistatic effects are the result of indirect genetic interactions explained by their common biochemical activities, common substrate interactions, and some expression overlap during vegetative growth. This is the first study to systematically examine redundancy in a family of genes in bacteria. Our results using the *B. subtilis* DBRHs as a case study, found no evidence of mutational robustness through functional compensation between duplicates, but rather interference among paralogs that evolved to function in interconnected pathways.

## Author Contributions

GO-Á, JG-G, and JS: conception or design of the study. DD-J, GO-Á, GG-S, JG-G, JS, and IV-P: acquisition, analysis, or interpretation of the data. DD-J, GO-Á, IV-P, JG-G, and JS: writing of the manuscript.

## Conflict of Interest Statement

The authors declare that the research was conducted in the absence of any commercial or financial relationships that could be construed as a potential conflict of interest.

## Footnotes

1 http://subtiwiki.uni-goettingen.de/
