# Supplementary material for: The DEAD-Box RNA Helicases of Bacillus subtilis as a Model to Evaluate Genetic Compensation Among Duplicate Genes
Source: Front Microbiol. 2018 Sep 25;9:2261. doi: 10.3389/fmicb.2018.02261 (PMC6178137; doi:10.3389/fmicb.2018.02261)
Supplement: Supplementary file 1 [file Data_Sheet_1.pdf]

José Antonio González-Gutiérrez[1], Diana Fabiola Díaz-Jiménez[1], Itzel Vargas-Pérez[1], Guillén-Solís, G[3,] Jörg Stülke[2], and Gabriela Olmedo-Álvarez[1,*]

*Corresponding author: Phone: +52 (462) 623 9600, Fax: +52 (462) 624 5846, E. mail: golmedo@ira.cinvestav.mx.

Supplementary Table S1. Oligonucleotides used in this study.

Primer	Sequence
**pX_deaD-F**	*GGTGGATCCTGAGAACATCTGCAAGAG
**pX_deaD-R**	*CCCGGATCCCACAGATGTCAGCCAGCA
**sigA-F**	GAAATTGCTGAGCGTATGTCCAGC
**sigA-R**	**CAGCTTGGTAGAATCGATCAGCTACCACTAATTAATTCAACACCTTGTTCACC
**gyrA-F**	ATCCATGGTCAGAATGGCTCAGG
**gyrA-R**	**CAGCTTGGTAGAATCGATCAGCTACTGAGTCTCCGTCAACAGAACCG
**cshA-F**	CGAATATTATTGTCGGAACACCTGG
**cshA-R**	**CAGCTTGGTAGAATCGATCAGCTACTCATCCGCTTCATCCATTACAACGG
**cshB-F**	GCAACTGACCTTGCCGCAAGAGG
**cshB-R**	**CAGCTTGGTAGAATCGATCAGCTACATCGCTTGTCCTGATGAGCCGG
**deaD-F**	CATCTGCCGACTGAGCGTACG
**deaD-R**	**CAGCTTGGTAGAATCGATCAGCTACTTTGACCTCGATATGCTCGGGG
**yfmL-F**	CTCAAAATGCATGAAGTGAAAACGATCG
**yfmL-R**	**CAGCTTGGTAGAATCGATCAGCTACGATTTGCTTCATCGTTTCGCGATGC
**Acopl**	**CAGCTTGGTAGAATCGATCAGCTAC
**QY-F**	***CCCCGACTAGTGCATGACGCAAACTTGGCCATTT
**QYA1-R**	CGTCATCGCCATACCTGTTTTTCCGGCCCGGCCTGTTCG
**QYA2-F**	GGACGAACAGGCCGGGCCGGAAAAACAGGTATGGCGATG
**QYA2-R**	*AGACCAGGATCCATCAAATTAGTAAGATTTTTTC
**QYB1-R**	GGTCATCGCTTGTCCTGATGATCCGGCCCGGCCTGTTCG
**QYB2-F**	GGACGAACAGGCCGGGCCGGATCATCAGGACAAGCGATG
**QYB2-R**	*CGTCCAGGATCCGGTTCCCCTACTTTCTTTTC
**QYD1-R**	CGAAATGGCCTTTCCTTTGTTTCCGGCCCGGCCTGTTCG
**QYD2-F**	GGACGAACAGGCCGGGCCGGAAACAAAGGAAAGGCCATT
**QYD2-R**	*GCTCCAGGATCCTCATTTATTCGCTTTATTC

* *Bam*H1 restriction sites are underlined. ** MYT4 sequences are underlined. *** *SpeI* restriction sites are underlined.

Supplementary Table S2. Differences of maximum and relative growth rate of simple and double mutants at 37°C.

	**Growth rate (r /h)**	**Relative growth (W)**
Wild type	0.97	1.00
ΔcshA	0.94	0.97
ΔcshB	0.84	0.87
ΔdeaD	0.92	0.95
ΔyfmL	0.92	0.95
ΔcshA::cat ΔcshB::mls	0.84	0.87
ΔcshA::cat ΔdeaD ::tet	0.92	0.94
ΔcshA::cat ΔyfmL::mls	0.93	0.96
ΔcshB::cat ΔdeaD ::tet	0.82	0.85
ΔcshB::cat ΔyfmL::mls	1.01	1.04
ΔdeaD::tet ΔyfmL::mls	1.00	1.03

Supplementary Table S3. Relative genetic expression of single mutant's background quantitated by qPCR using *sigA* as control gene.

	ΔcshA	***ΔcshB***	***ΔdeaD***	***ΔyfmL***
cshA	0.2 ± 0.23	2.1± 0.26	4.0 ± 0.85	5.5 ± 0.33
cshB	22.5 ± 0.25	0.0 ± 0.58	1.3 ± 0.91	2.8 ± 0.12
deaD	4.4 ± 0.41	5.1± 0.40	0.0 ± 0.29	6.9 ± 0.14
yfmL	1.3 ± 0.40	2.2 ± 0.21	2.7 ± 0.53	0.0 ± 0.45

Mean and SD, standard deviation of three independent experiments with triplicate

Supplementary Table S4. Growth rate of self-complementation strains at 18°C.

	**Growth rate (r / h) without Inducer**	**Growth rate (r/ h) with inducer**
Wild type	0.27	0.27
ΔcshA pxylA::cshA	0.09	0.23
ΔcshB pxylA::cshB	0.17	0.25
ΔdeaD pxylA::deaD	0.26	0.27
ΔyfmL pxylA::yfmL	0.18	0.23

Mean of duplicate data

Supplementary Table S5. Cross complementation of DEAD-Box RNA helicase mutants

	**Control***	***PxylA::cshA***	***PxylA::cshB***	***PxylA::deaD***	***PxylA::yfmL***
ΔcshA					
**(-)**	0.103 ± 0.006	.09	0.062 ± 0.006	0.081 ± 0.003	0.077 ± 0.006
**(+)**	0.105 ± 0.006	0.23	0.072 ± 0.002	0.092 ± 0.003	0.084 ± 0.004
**ΔcshB**					
**(-)**	0.168 ± 0.004	0.166 ± 0.011	.17	0.202 ± 0.009	0.222 ± 0.011
**(+)**	0.174 ± 0.005	0.153 ± 0.005	.25	0.175 ± 0.008	0.215 ± 0.016
ΔdeaD					
**(-)**	0.371 ± 0.012	0.376 ± 0.015	0.308 ± 0.007	.26	0.321 ± 0.009
**(+)**	0.380 ± 0.009	0.375 ± 0.000	0.307 ± 0.010	.27	0.313 ± 0.016
ΔyfmL					
**(-)**	0.130 ± 0.002	0.137 ± 0.002	0.142 ± 0.003	0.143 ± 0.007	.18
**(+)**	0.129 ± 0.002	0.119 ± 0.002	0.132 ± 0.003	0.137 ± 0.007	.23

Maximum growth rate **at 18ºC**, obtained with logistic model fit. Without xylose as an inducer (-) with xylose 1% (+). * Parental strains without ectopic construction for gene expression. Mean and SD, standard deviation of triplicate sample.

Supplementary Table S6. RecA-like domain identity/similarity

	**CshA**	**CshB**	**DeaD (YxiN)**	**YfmL**
**CshA**	-			
**CshB**	67.8/84.7	-		
**DeaD (YxiN)**	71.9/90.4	67/89.6	-	
**YfmL**	58.4/87.6	61.9/85	57.5/87.6	-
