# Supplementary material for: The DEAD-Box RNA Helicases of Bacillus subtilis as a Model to Evaluate Genetic Compensation Among Duplicate Genes
Source: Front Microbiol. 2018 Sep 25;9:2261. doi: 10.3389/fmicb.2018.02261 (PMC6178137; doi:10.3389/fmicb.2018.02261)
Supplement: Supplementary file 2 [file Data_Sheet_2.pdf]

José Antonio González-Gutiérrez[1], Diana Fabiola Díaz-Jiménez[1], Itzel Vargas-Pérez[1], Guillén-Solís, G[3,] Jörg Stülke[2], and Gabriela Olmedo-Álvarez[1,*]

*Corresponding author: Phone: +52 (462) 623 9600, Fax: +52 (462) 624 5846, E. mail: golmedo@ira.cinvestav.mx.

Supplementary Figures

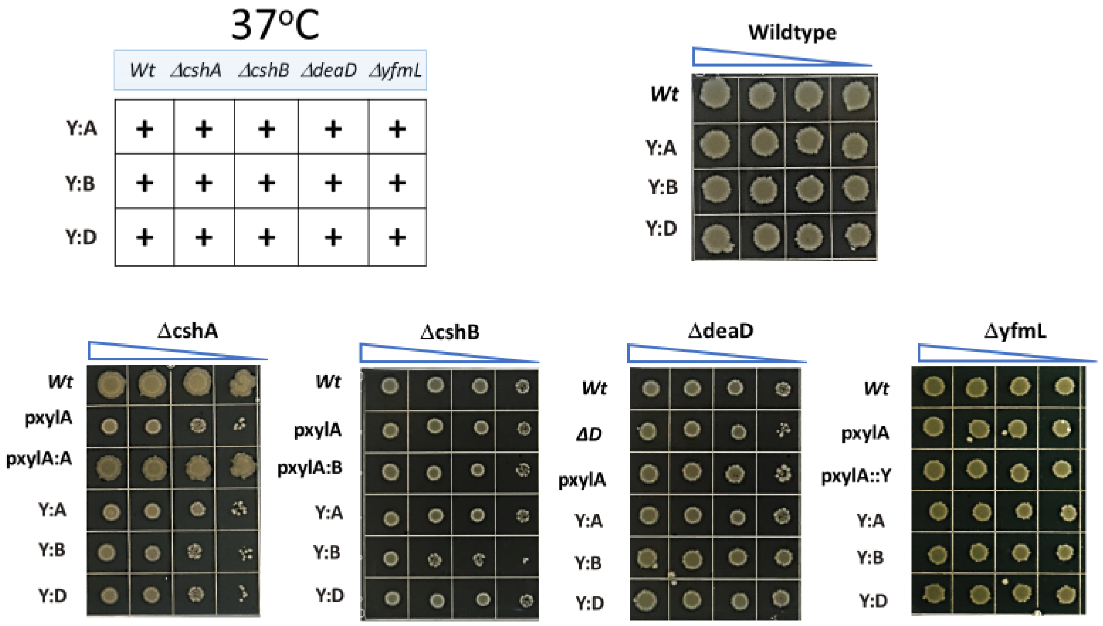

Figure S1. In-vivo functional analysis of chimeric helicases. The expression of chimeric helicases was driven by the xylose inductor and growth was tested in the wild-type and each mutant background. Strains were growth and dilutions were spotted onto LB-agar and incubated at 37°C . (+) means optimal growth.

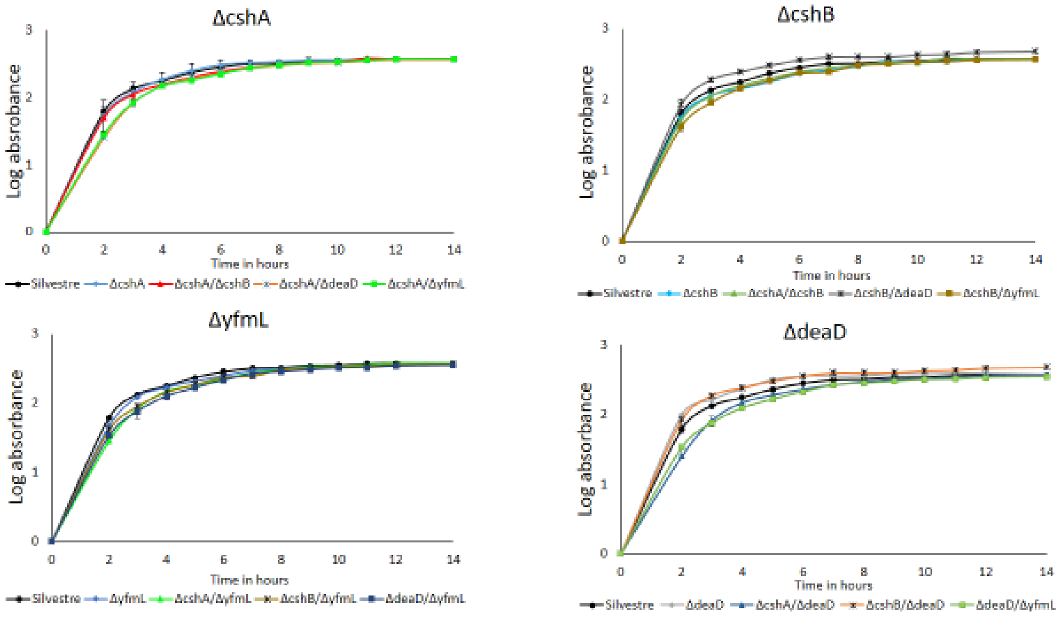

Figure S2. Growth of single and double mutants at 37 °C. Points are triplicate mean and bars represent standard deviation. The samples were incubated in LB broth at 37 °C.

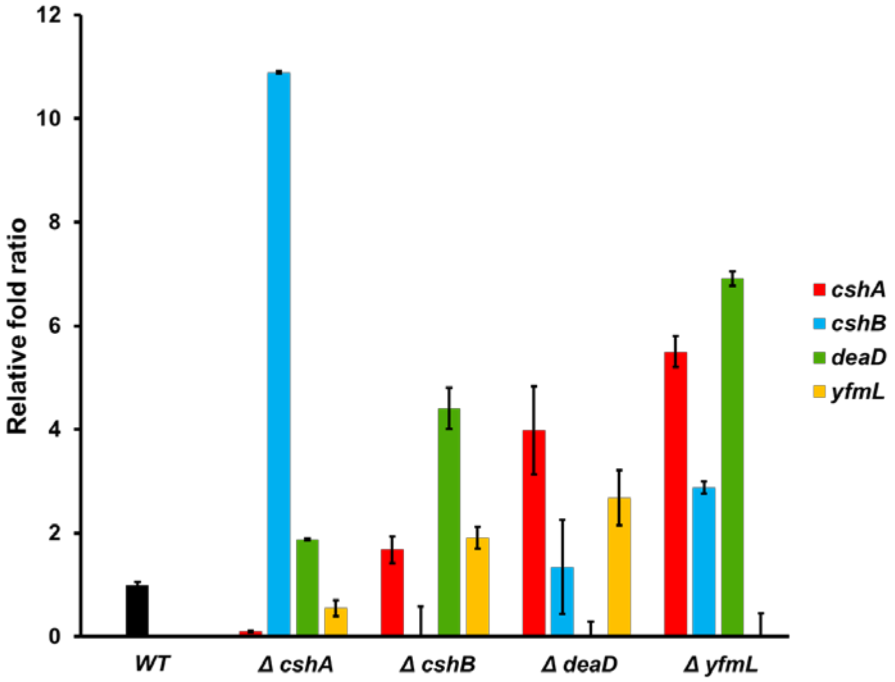

Figure S3. Differential effect in genetic expression when a single RNA helicase gene is deleted at 18 °C. Relative expression quantification was performed by real-time PCR from nucleic acids extract in exponential phase of *cshA* (GP 1083), *cshB* (GP1051), *deaD (YxiN)* (GP 1052) and *yfmL* (GP1053) mutants backgrounds. The bars represent the standard deviation of three independent experiments. The *sigA* gene was used for expression normalization and the wild-type was the comparative biological sample.

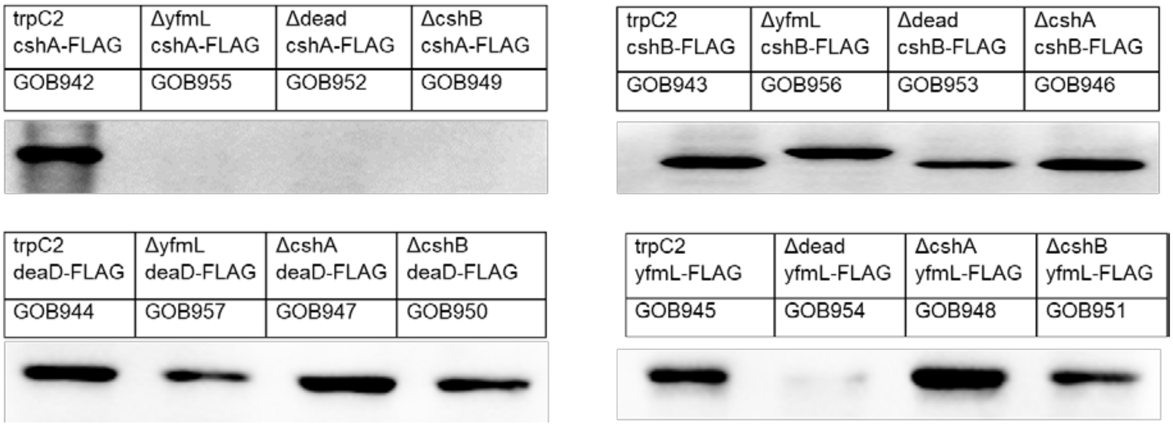

Figure S4. Protein expression of FLAG-tagged DEAD-Box RNA helicases in different mutant backgrounds. Proteins extracted during exponential phase at 18 ^{o}C were detected with anti-FLAG monoclonal antibody. Western blot was performed in wild-type and single mutants' background.

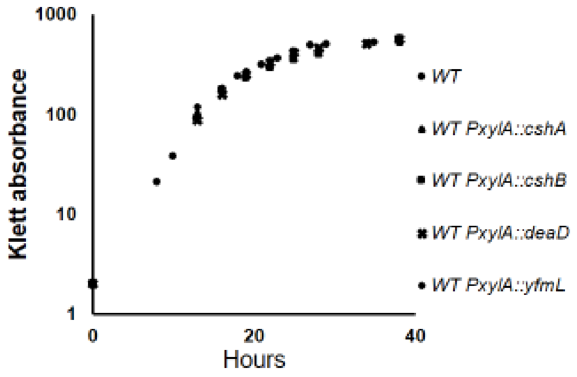

	Growth rate (r)
Wt	0.28 ± 0.006 ^{a}
Wt PxylA::cshA	0.25 ± 0.002 ^{b}
Wt PxylA::cshB	0.25 ± 0.013 ^{b}
Wt PxylA::deaD	0.26 + 0.005 ^{ab}
Wt PxylA::yfmL	0.26 ± 0.010 ^{ab}

Figure S5. Discrete dose effect in wild-type phenotype at overexpressing RNA helicase genes. The strains GOB 642, GOB 643, GOB 644, GOB 645 were incubated at 18 °C with inducer (xylose 1%). The table shows the values calculated by the logistic model.

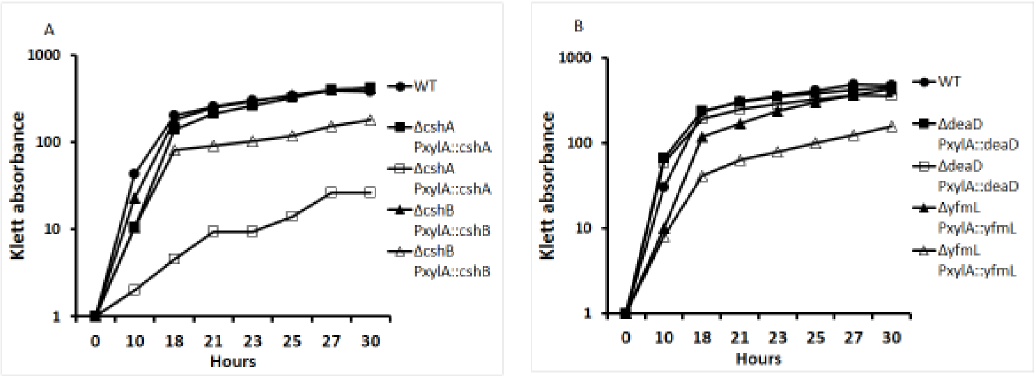

Figure S6. Auto-complementation is observed in the presence of 1 % inducer. Strains GP1084, GP 1086, GP 1087, GOB 627, were incubated in LB medium without A) and with B) inducer (xylose) at 18°C.

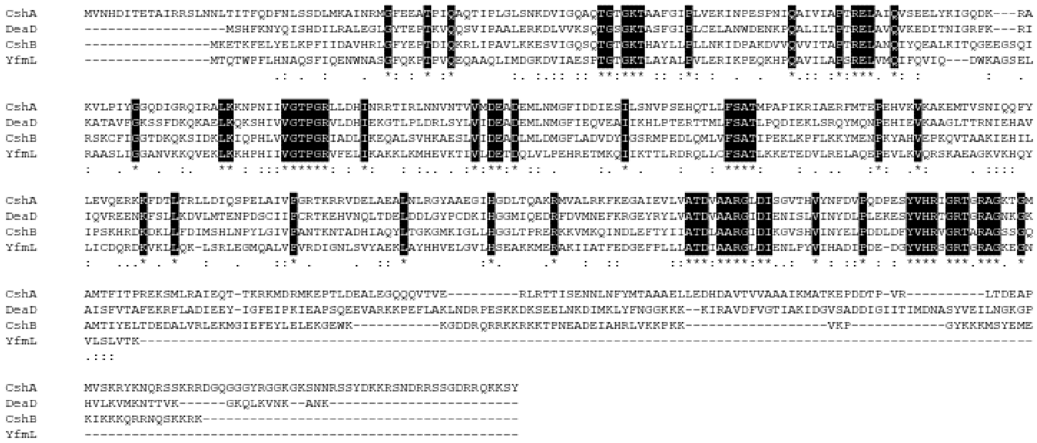

DBRH	Length C' end[a]	pI	Lys	Arg	Glu	Asp	Gly	Leu
CshA	158	10.03	10.1	11.4	7	7	6.3	5.1
CshB	98	10.03	23.5	9.2	12.2	5.1	5.1	6.1
DeaD	144	9.52	19.7	4.4	7.6	5.7	6.4	5.1
YfmL	41	9.76	24.4	0	12.2	0	9.8	17.1

Figure S7. Alignment of the four *B. subtilis* RNA helicase protein sequences. Amino acid sequences were obtained in SubtiWiki 2.0--an integrated database for the model organism *Bacillus subtilis* (Michna et al., 2016), and aligned using Clustal Omega (EMBL-EBI). The consensus sequence is highlighted in black. The table shows per cent content of charged amino acids and calculated Isoelectric point. Only values above 9% are shown. Data were obtained using an online tool https://web.expasy.org/protparam/ Bold numbers emphasize values that are 9% or higher.
